# Improved Active Disturbance Rejection Double Closed-Loop Control of a Rotary-Type VCM in a Moving Mirror Control System

**DOI:** 10.3390/s22103897

**Published:** 2022-05-20

**Authors:** Liangjie Zhi, Min Huang, Wei Han, Zhanchao Wang, Xiangning Lu, Yang Bai, Han Gao

**Affiliations:** 1 Aerospace Information Research Institute, Chinese Academy of Sciences, No.9 Dengzhuang South Road, Haidian District, Beijing 100094, China; zhiliangjie20@mails.ucas.ac.cn (L.Z.); hw@aircas.ac.cn (W.H.); wangzhanchao@aoe.ac.cn (Z.W.); luxn@aircas.ac.cn (X.L.); baiyang171@mails.ucas.ac.cn (Y.B.); gaohan@aircas.ac.cn (H.G.); 2 School of Optoelectronics, University of Chinese of Academy Sciences, No.19(A) Yuquan Road, Shijingshan District, Beijing 100049, China; 3 Department of Key Laboratory of Computational Optical Imagine Technology, CAS, No.9 Dengzhuang South Road, Haidian District, Beijing 100094, China

**Keywords:** moving mirror control system, VCM, active disturbance rejection controller, improved extended state observer

## Abstract

Aiming to address the problem of moving mirror speed fluctuations in moving mirror control systems, an improved active disturbance rejection double closed-loop controller (IADR-DCLC) is proposed and verified by simulation to realize the high-performance control of a moving mirror control system. First, the mathematical model of a rotary-type voice coil motor (RT VCM) is established, and the relationship between the angular velocity of the RT VCM and the optical path scanning velocity is analyzed. Second, in order to suppress the model uncertainty and external disturbance of the system, an improved active disturbance rejection controller (IADRC) is proposed. Compared with a conventional ADRC, the tracking differentiator of the proposed IADRC is replaced with desired signal optimization (DSO), and the actual speed is introduced to the extended state observer (ESO). The IADRC is used in the position–speed double closed-loop control model. Finally, the simulation results show that the IADR-DCLC has not only a good tracking effect but also a good anti-interference ability and can meet the requirements of the moving mirror control system for the uniformity of optical-path scanning speed and accurate control of the position of the moving mirror.

## 1. Introduction

In recent years, with the rapid development of electronics, spectroscopy, and computer technology, Fourier transform infrared (FTIR) spectrometry is widely used in quantitative and qualitative analyses of chemical substances. The Fourier transform infrared spectrometer is a fast, wide spectral band, high-resolution, and high signal-to-noise ratio detection tool that can realize the rapid qualitative and quantitative analyses of solid, liquid, powder, and viscous samples [1,2,3,4]. A moving mirror is the core component of the Fourier transform infrared spectrometer. The stability of the scanning speed of the moving mirror is very important. The scanning speed of the moving mirror determines the frequency of the interferogram. The instability of any frequency in the interferogram will cause additional peaks in the Fourier transform spectrum and affect the spectral restoration [5]. Therefore, the moving mirror control system requires not only the moving mirror to move at a uniform speed but also the position of the moving mirror to be accurate [6,7]. At present, the mainstream Fourier transform spectrometer mostly uses a VCM to drive the moving mirror [8]. VCMs have the advantages of small volume, large thrust, fast response, and high precision. Through algorithm control, they can complete the high-precision servo control task of the micron and even nanometer level [9].

However, the VCM is vulnerable to the following factors: (1) external environmental factors, such as temperature and magnetic field—the external environment can affect the parameters of the VCM, such as coil resistance and magnetic induction intensity, which makes the mathematical model of the VCM difficult to accurately describe; (2) the mechanical characteristics of VCM, such as friction characteristics—the VCM will show different friction characteristics at different motion stages. The interference of the external environment and the influence of the mechanical characteristics of VCMs aggravate their nonlinear characteristics, seriously affect their positioning accuracy and speed control, and produce serious challenges for the design of high-performance moving mirror control systems [10]. Therefore, research of a control algorithm that can compensate for the nonlinear disturbance and overcome the problem that the model of the VCM cannot accurately describe is of great significance to the high-performance moving mirror control system.

To obtain a high-performance moving mirror control system, scholars have designed many control algorithms to improve the stability of the optical-path scanning speed and overcome the influence of disturbance on the VCM. Dou X. (2008) adopted the fuzzy PID control algorithm to ensure the moving mirror reaches the uniform motion stage within 0.1 s, which shortens the response time of the system [11]. Shi Y. (2011) designed a fuzzy integral sliding mode variable structure control for the RT VCM, which effectively suppresses the interference and chattering of the system and has high-speed tracking stability [12]. Zhang M. et al. (2014) chose the VCM to directly drive the moving mirror and used the nonlinear PID controller based on the disturbance observer to control the speed of the moving mirror, which performed significantly better than the conventional PID [13]. However, it is easy to limit the design of a low-pass filter for the disturbance observer. Li T. et al. (2014) proposed a PID control algorithm adjusted by an adaptive online genetic algorithm that can better suppress the added artificial disturbance, but the algorithm is complex [14]. Xia X. (2014) proposed a method to measure the moving mirror speed for the slow motion of the moving mirror, which improved the motion accuracy of the slow motion of the moving mirror [15]. Shi L. et al. (2015) added performance evaluation rules to the PID control system, which can select different PID parameters according to the performance evaluation rules. Through the adjustment of the control algorithm parameters, the scanning accuracy of the moving mirror mechanism was improved [16]. However, it is difficult to determine the fuzzy rules and evaluation rules in the literature [11,12,16], and it can only take a limited level, which limits the control accuracy. However, the tracking accuracy needs to be improved. Guo L. et al. (2020) designed the speed feedback loop and the position loop of a PI controller for the controlled object and inserted the repetitive controller into the control system to improve the control accuracy and effectively suppress the periodic disturbance [17]. However, the repetitive controller needs to run multiple cycles to reduce the error. It can be seen from the above literature that the nonlinear disturbance in the moving mirror control system is the main factor affecting the stability of the optical-path scanning velocity. Therefore, the focus of this paper is to find an effective control strategy to compensate for the disturbance and improve the performance of the system.

Han Jing-Qing proposed an active disturbance rejection control theory [18,19]. This method does not depend on the accurate mathematical model of the controlled object. It can estimate and compensate for the interference inside and outside the system in real-time. It has the advantages of a simple algorithm, strong robustness, fast system response, and strong anti-interference ability. The ADRC can use the extended state observer to observe the state of the system and the total disturbance inside and outside the system and compensate for the disturbance of the system input through the feedforward method to linearize the system [20]. At present, the ADRC has been widely used in the field of motor control [21]. The conventional ADRC only uses the position feedback and does not process the instantaneous speed of the measured position [22], so the performance of the speed control is slightly unsatisfactory. Aiming at the characteristic of the moving mirror control system to calculate the real optical-path scanning speed according to the frequency of interferogram, the IADRC is proposed in this paper, and it is used in the position–speed double closed-loop control algorithm. The position loop of the algorithm adopts the proposed IADRC, and the speed loop adopts the conventional PI control algorithm. The IADR-DCLC proposed in this paper can still possess a good control effect when the parameters of the controlled object change or are disturbed by external disturbance, and it has good anti-disturbance ability that can meet the requirements of the high precision and anti-interference of the moving mirror control system.

The rest of this paper is organized as follows: Section 2 mainly introduces the principles and methods. Firstly, this section presents an analysis of the principle of the moving mirror control system, establishes the mathematical model of the RT VCM, and theoretically presents an analysis of the relationship between the angular speed of the RT VCM and the optical-path scanning speed. Secondly, based on the above-mentioned related model research, in this study, we planned the ideal motion trajectory of the moving mirror. Finally, the IADRC was designed. Section 3 focuses on the simulation results and analysis. In this section, the control model of the RT VCM established in MATLAB is presented along with the simulation algorithm designed to simulate the controller proposed in this paper. The conclusions of this paper are provided in Section 4.

## 2. Principles and Methods

### 2.1. Mathematical Model of RT VCM

The RT VCM is the core component of a moving mirror control system, and its performance affects the quality of the spectrum. Compared to the stepping motor and finite angle motor, the RT VCM has the advantages of small volume, good control characteristics, and outstanding performance advantages [9]. Based on the performance advantages of the RT VCM, in this study, we chose it as the core component of the moving mirror control system, meeting the requirements of the response speed and steady-state accuracy of the system.

**Figure 1:**
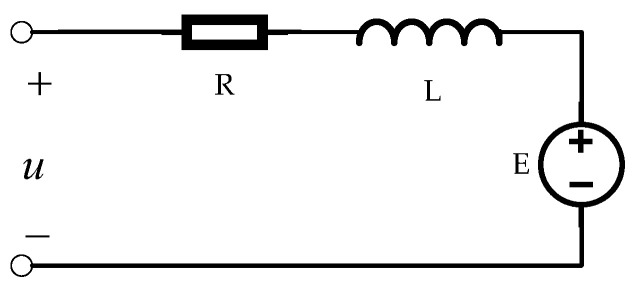
The electrical model.

The electrical model of the RT VCM under ideal conditions is shown in Figure 1. The relationship between the voltage and back EMF can be obtained [23], and the electrical balance equation can be listed as follows:

(1) $$u=E+iR+L\frac{di}{dt}$$

where u is the voltage of the motor armature, E is the induced electromotive force of the motor, i is the current of the motor armature, L is the inductance of the motor armature, and R is the resistance of the motor armature. The induced electromotive force of the motor is directly proportional to the angular velocity of the motor [23], and the equation is as follows:

(2) $$E=B_{g}L_{e}\omega =B_{g}L_{e}\frac{d\theta }{dt}=k_{a}\frac{d\theta }{dt}$$

where $B_{g}$ is the electromagnetic strength of the RT VCM, $L_{e}$ is the effective length of the coil of the RT VCM, $k_{a}$ is the motor torque coefficient, ω is the angular velocity of the motor rotor, and θ is the angular displacement of the RT VCM.

The swing arm with the angled mirror is connected to the VCM through the elastic hub. The mechanical balance formula of the RT VCM with a load can be obtained through the mechanical principle as follows [24]:

(3) $$\begin{array}{cc} F=B_{g}L_{e}i & =k_{a}i=(F_{m}+F_{r})+F_{c}+F_{z}\\ & =J_{sum}\frac{d^{2}\theta }{dt^{2}}+k\frac{d\theta }{dt}+b\theta \end{array}$$

where $F_{m}$ is the inertia force determined by the mass of the rotor of the RT VCM and its acceleration, $F_{r}$ is the inertia force determined by the mass of the load and its acceleration, $F_{c}$ is the dynamic friction between the machinery, $F_{z}$ is the elastic force generated at the connection between the load and the motor, $J_{sum}$ is the total moment of inertia between the rotor and the load, k is the friction damping coefficient, and b is the elastic damping coefficient.

Equations (4) and (5) can be obtained by the Laplace transform of Equations (1) and (3), respectively:

(4) $$\begin{array}{ll} U(s) & =E(s)+Ri(s)+Lsi(s)\\ & =k_{a}s\theta (s)+Ri(s)+Lsi(s) \end{array}$$

(5) $$k_{a}i(s)=J_{sum}s^{2}\theta (s)+ks\theta (s)+b\theta (s)$$

The transfer function G(s) between the angular displacement and the voltage of the RT VCM can be obtained by combining Equations (4) and (5):

(6) $$G(s)=\frac{\theta (s)}{U(s)}=\frac{k_{a}}{LJ_{\mathrm{sum}}s^{3}+(J_{sum}R+Lk)s^{2}+({k_{a}}^{2}+kR+bL)s+bR}$$

**Figure 2:**
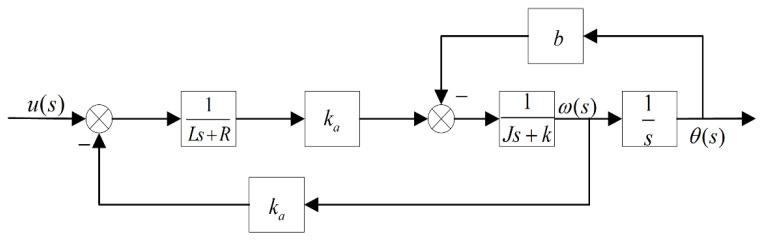
A mathematical model block diagram of the RT VCM.

According to G(s), the mathematical model block diagram of the RT VCM can be obtained as shown in Figure 2.

### 2.2. The Relationship between the Optical Path Scanning Speed and the Angular Speed of the RT VCM

Assuming that the frequency of the incident light is σ and the optical path scanning speed of the FTIR based on the Michelson interference principle is v, the frequency of interferogram generated by the FTIR modulation of the incident light is as follows [25]:

(7) f=2vσ

If the moving speed of the moving mirror cannot be controlled stably, the optical-path scanning speed will fluctuate, which will change the frequency of the interferogram generated by FTIR and affect the quality of the spectrum retrieved from the interferogram [26,27]. According to the literature review by [28], when the mean square error of the optical-path scanning speed is less than 2%, the impact of the moving mirror speed fluctuation on the quality the of the spectrum can be ignored.

**Figure 3:**
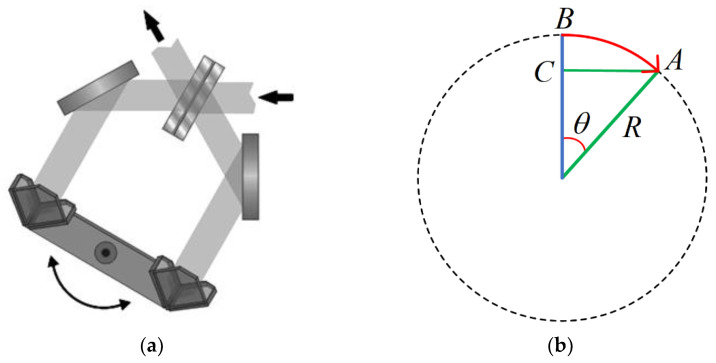
(**a**) The double moving mirror torsion pendulum structure. (**b**) A schematic diagram of the relationship between the OPD and the swing angle.

For the double moving mirror torsion pendulum optical structure, shown in Figure 3a, the change in the OPD can be simplified as shown in Figure 3b [29]. Assuming point *B* is the starting center position of the swing arm, and swinging the swing arm θ radians to point A, the OPD generated by this motion can be regarded as *AC*, that is, AC=Rsinθ. At the same time, according to the optical-path design in Figure 3, the light needs to enter and exit the angled mirror twice, and both sides of the swing arm are symmetrical. With the rotation of the swing arm, the OPD of the whole interference system changes as follows:

(8) x=4Rsinθ

where R is the arm length of the swing arm, x is the OPD, θ is the rotation angle of the swing arm, and is also the rotation angle of the RT VCM. Taking the derivative with respect to t in Equation (8),

(9) v=4Rωcosθ

where v is the optical path scanning speed, and ω is the angular velocity of the swing arm, which is also the angular velocity of the RT VCM. When the swing range of the swing arm is small, the Taylor expansion of cosθ can be approximately obtained as follows:

(10) $$\omega \approx \frac{v}{4R}(1+\frac{\theta^{2}}{2})$$

Equation (11) is obtained by solving a differential Equation (10):

(11) $$\{\begin{array}{l} \theta =\sqrt{2}tg(\frac{\sqrt{2}v}{8R}t)\\ \omega =\frac{v}{4R}\sec^{2}(\frac{\sqrt{2}v}{8R}t) \end{array}$$

**Figure 4:**
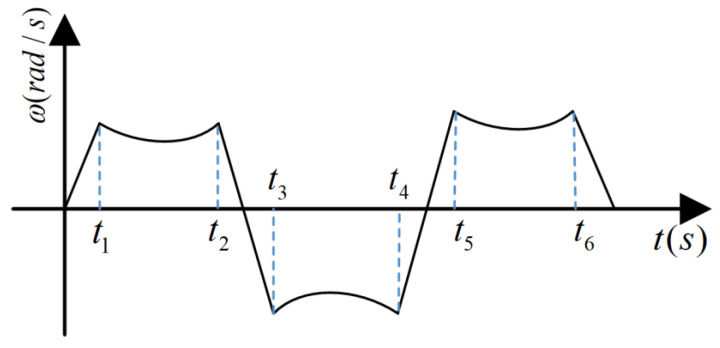
The relationship between the angular velocity and the time of the RT VCM.

It can be seen from Equation (11) that the motor angular speed of the moving mirror control system is a time-varying relationship with the optical-path scanning speed; that is, given a fixed optical-path scanning speed, the system requires the realization of a time-varying angular speed of the RT VCM. The time-varying relationship is shown in Figure 4 [12].

Combined with sinusoidal commutation, in this study, we designed the speed curve of the RT VCM such that the angular speed curve was as smooth as possible.

**Figure 5:**
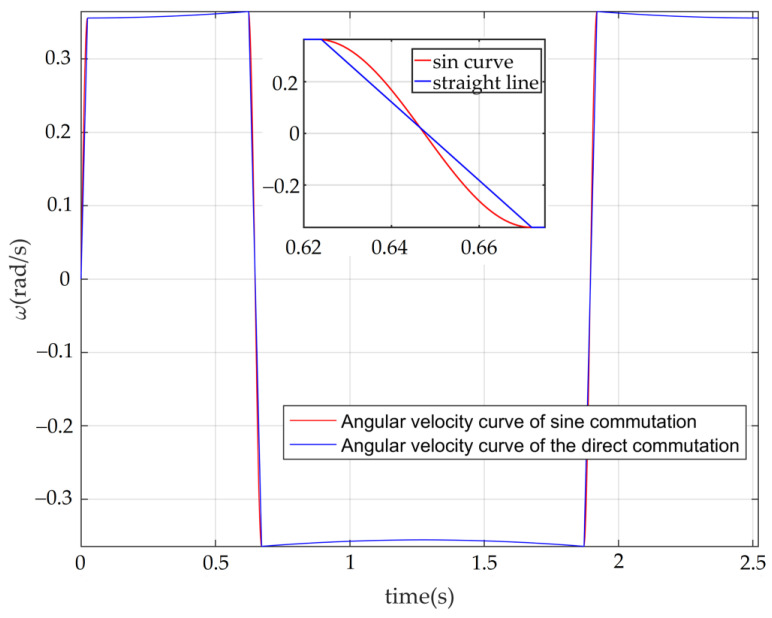
The angular velocity curve of the RT VCM.

**Figure 6:**
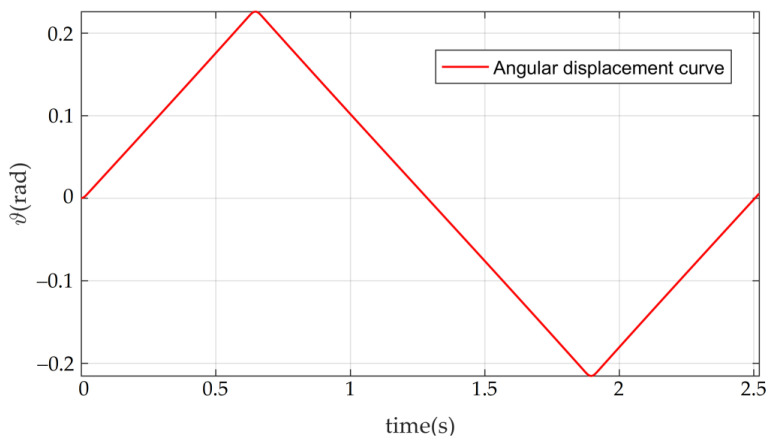
The angular displacement curve of the RT VCM.

According to the performance parameters in Table 1, the relationship between the angular speed and the time of the RT VCM finally determined is shown in the red curve in Figure 5, in which the blue curve represents the angular speed without sinusoidal commutation. The relationship between the angular displacement and the time corresponding to the angular velocity is shown in Figure 6.

### 2.3. Design of the Improved Active Disturbance Rejection Double Closed-Loop Controller

In order to ensure that the speed and position of the moving mirror meet the interference requirements, we adopted the position-speed double closed-loop control method to ensure the stability of the optical-path scanning speed and to accurately control the angular displacement of the RT VCM [30]. The IADRC was adopted for the position loop, and the conventional PI control algorithm was adopted for the speed loop.

**Figure 7:**
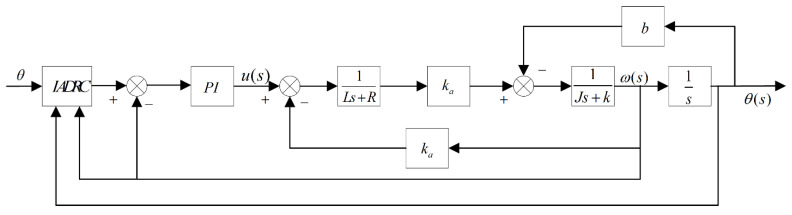
The overall block diagram of the control system.

The control structure block diagram, after connecting the position loop and the speed loop in the series, is shown in Figure 7. The input of the position loop is the given angular displacement signal θ, and the output is the input of the speed loop. After the calculation of the speed loop, the output voltage signal drives the RT VCM [31].

#### 2.3.1. The Principle of IADRC

**Figure 8:**
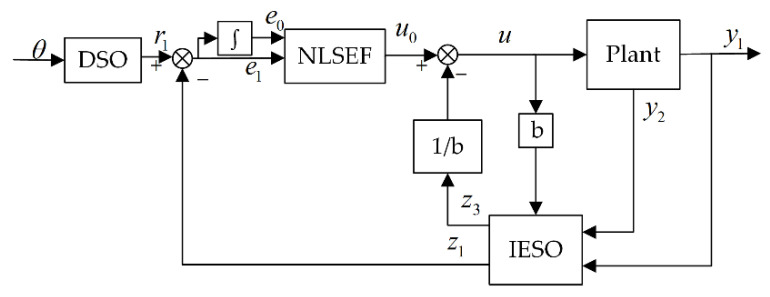
The improved active disturbance rejection control framework.

Compared to the conventional ADRC [22], the IADRC reduces the response time of the controller and effectively reduces the impact of the input signal on the system by designing the desired signal in advance and avoiding the use of a tracking differentiator [32]. The extended state observer (ESO) is improved by inputting the actual speed value into the observer so that the observed speed is closer to the actual speed and the disturbance is concentrated on the disturbance observation value of the observer. The proposed improved ADRC is shown in Figure 8 and includes three modules: the desired signal optimization (DSO), the improved extended state observer (IESO), and the nonlinear state error feedback (NLSEF).

##### Desired Signal Optimization (DSO)

In the moving mirror control system, the motion curve of the RT VCM can be designed in advance, that is, the desired motion curve design presented in Section 2.2. Therefore, the differential signal can be calculated through a tracking differentiator in advance to reduce the response time of the controller. At the same time, the impact of the shocks can be reduced to improve the stability of the tracking process.

##### Improved Extended State Observer (IESO)

In this control system, the actual speed can be measured so that the value of the actual speed can be used to bring the observer $z_{2}(t)$ closer to $r_{2}(t)$ to concentrate the system disturbance more on $z_{3}(t)$ and to improve the estimation accuracy of the disturbance [22]. The third-order equation of IESO is as follows:

(12) $$\{\begin{array}{l} \begin{array}{l} e_{1}=z_{1}-y_{1}\\ e_{2}=z_{2}-y_{2} \end{array}\\ {\dot{z}}_{1}=z_{2}-\beta_{1}e_{1}\\ {\dot{z}}_{2}=z_{3}-\beta_{2}e_{2}+bu\\ {\dot{z}}_{3}=-\beta_{3}fal(e_{1},\alpha ,\delta ) \end{array}$$

(13) $$fal(e,\alpha ,\delta )=\{\begin{array}{ll} \frac{e}{\delta^{1-\alpha }} & \left|e\right|\leq \delta \\ {\left|e\right|}^{\alpha }\mathrm{sgn}(e) & \left|e\right|>\delta \end{array}$$

where $z_{1}(t)$ is going to be close to $r_{1}(t)$; $z_{2}(t)$ is going to be close to $r_{2}(t)$; $z_{3}(t)$ is used to estimate the uncertainty and external interference of the model; $\beta_{1}$, $\beta_{2}$, and $\beta_{3}$ are the observation gains; α and δ are the design parameters; and b is a constant. The nonlinear function fal(•) has the characteristics of a large gain when the error is small and a small gain when the error is large, which can improve the dynamic performance of the system [22].

##### Nonlinear State Error Feedback (NLSEF)

In this study, a nonlinear combination in PI form was used [24], which can be expressed as follows:

(14) $$u_{0}=\beta_{01}fal(e_{0},\alpha_{01},\delta )+\beta_{02}fal(e_{1},\alpha_{02},\delta )$$

(15) $$u=z_{3}-\frac{u_{0}}{b}$$

where $e_{0}$ and $e_{1}$ represent the integral of the error and the error, respectively, and $\beta_{01}$, $\beta_{02}$, $\alpha_{01}$, and $\alpha_{02}$ are the parameters of the controller.

#### 2.3.2. PI Controller of Speed Loop

In the moving mirror control system, the steady-state velocity error of the OPD velocity is required to be as small as possible and the response speed of the speed loop is fast. In this study, the speed loop adopted the PI controller, which can achieve an ideal control effect [33]. The digital PI control algorithm can be expressed as follows:

(16) $$u(k)=k_{P}e(k)+k_{1}\sum_{i=0}^{k}e(i)$$

where u(k) is the duty cycle calculated at the moment; $k_{P}$ and $k_{1}$ are the proportional coefficient and the integral coefficient, respectively; and e(k) is the error at the moment.

## 3. Simulation Results and Analysis

### 3.1. Parameter Settings

The parameters of the moving mirror control system are shown in Table 2.

Based on the above parameters, we used Simulink to design the IADR-DCLC. We also designed the classical PI double closed-loop controller, the fuzzy PI double closed-loop controller, the conventional active disturbance rejection double closed-loop controller, and the PI double closed-loop controller based on disturbance observer (DOB). We determined the parameters of the five controllers by adjusting them.

The integration step in the simulation was 0.001, and the parameters of the IESO in the IADR-DCLC were $\beta_{1}=2000$, $\beta_{2}=1500$, $\beta_{3}=10,000$, α=0.75, δ=0.001, and b=6. The parameters of the NLSEF were $\beta_{01}=40$, $\beta_{02}=4$, $\alpha_{01}=0.6$, $\alpha_{02}=0.4$, and δ=0.002. The parameters of the speed loop were $k_{P}=200$, $k_{1}=1000$. In this paper, the parameters of the speed loop of the five controllers were the same. The parameters of the position loop of the classical PI double closed-loop controller were $k_{P}=100$, $k_{1}=1000$. Based on the classical PI double closed-loop controller, this paper designed a 3 × 3 fuzzy rule table for the fuzzy PI double closed-loop controller. The common parameters of the conventional active disturbance rejection double closed-loop controller and the IADR-DCLC were the same. Only in ESO, the two parameters were different. The two parameters were $\alpha_{1}=0.45$, $\alpha_{2}=0.55$. For PI the double closed-loop controller based on DOB, the low-pass filter designed in this paper was

(17) $$Q(s)=\frac{2\tau s+1}{\tau^{2}s^{2}+2\tau s+1}$$

where τ determines the bandwidth of Q(s). In this paper, the value of τ was 0.01. The parameters of the position loop of PI double closed-loop controller based on DOB were $k_{P}=220$, $k_{1}=2000$.

### 3.2. Simulation Results

To verify the performance of this controller, we carried out a simulation analysis of the controller in three situations. Firstly, the steady-state performance and dynamic performance of each controller were compared by observing the step response of these controllers to verify the good performance of the controller. Then, the desired signal was input into these controllers to compare the performance of each controller to verify whether the controller can meet the requirements of the moving mirror control system. Finally, the time-varying disturbance was added to these controllers to test and compare the anti-jamming performance and the robustness of each controller.

#### 3.2.1. Step Response

**Figure 9:**
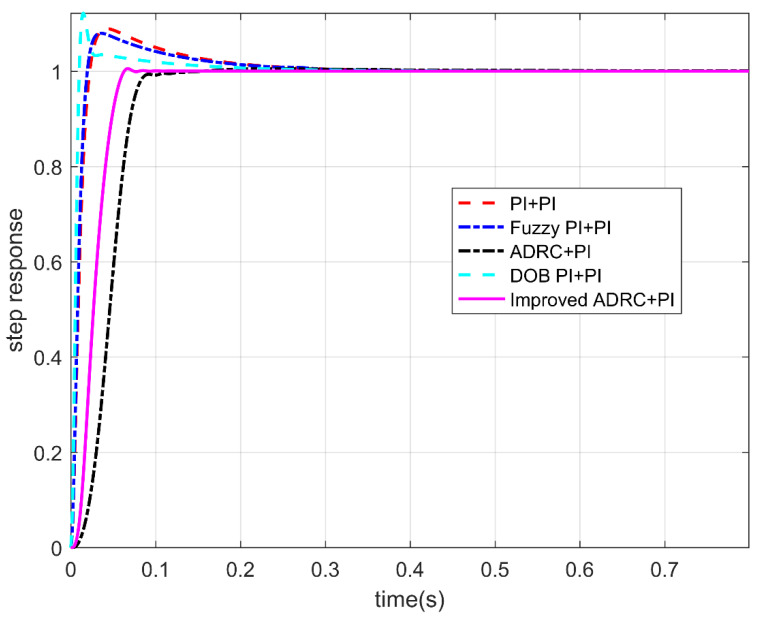
The step response of five controllers.

The step responses of the five controllers when the controller input is a step signal are shown in Figure 9, and the step response indexes are shown in Table 3. According to Figure 9 and Table 3, it can be concluded that when the input is a step signal, the classical PI double closed-loop controller, the fuzzy PI double closed-loop controller, and the PI double closed-loop controller based on DOB have a large overshoot. The overshoot of the conventional active disturbance rejection double closed-loop controller and the IADR-DCLC is almost zero. Compared to the conventional active disturbance rejection double closed-loop controller, the IADR-DCLC causes the system to enter the steady state faster. In addition, the final steady-state error of the IADR-DCLC is 0%. The IADR-DCLC shows excellent dynamic performance and steady-state performance.

#### 3.2.2. Simulation of Controllers without Disturbance

**Figure 10:**
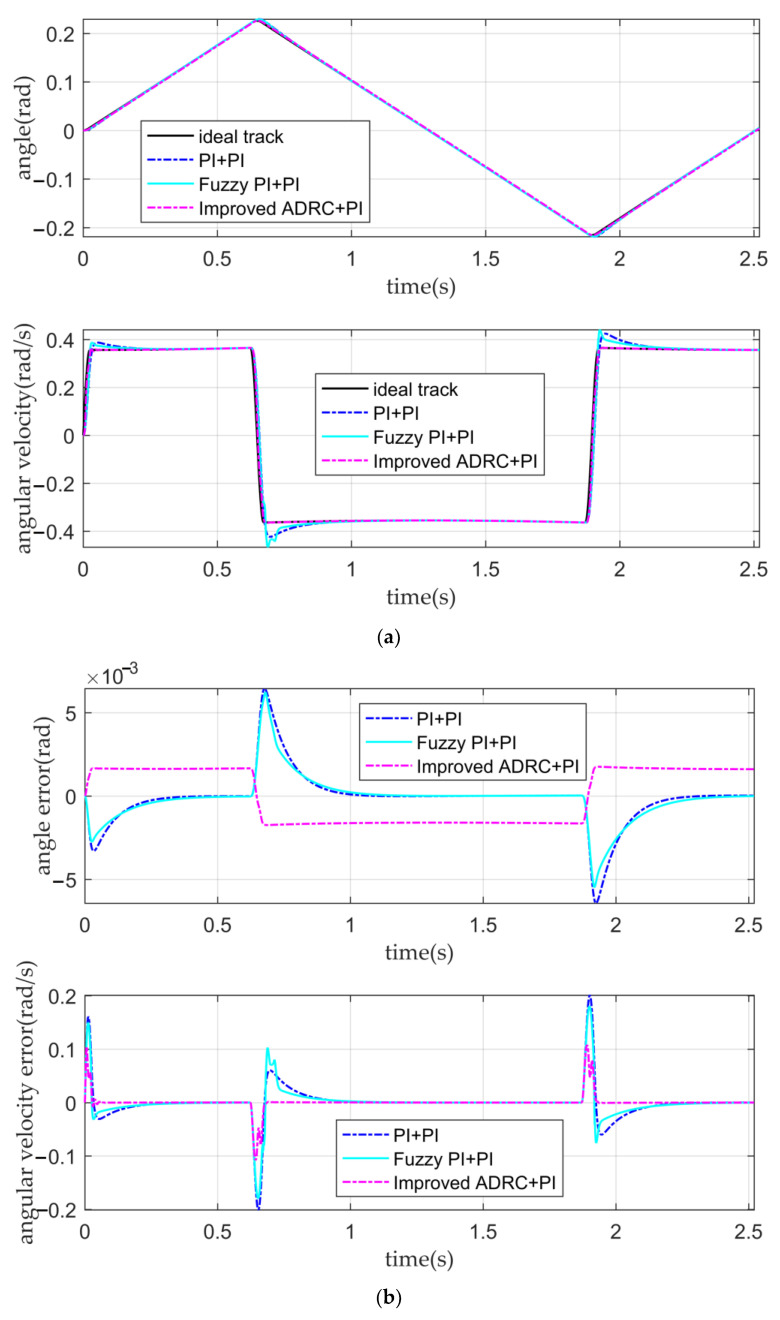
The tracking curves and the error curves. (**a**) The angular displacement tracking curves and the angular velocity tracking curves of the classical PI double closed-loop controller, the fuzzy PI double closed-loop controller, and the IADR-DCLC; (**b**) the angular displacement error curves and the angular velocity error curves of the classical PI double closed-loop controller, the fuzzy PI double closed-loop controller, and the IADR-DCLC.

**Figure 11:**
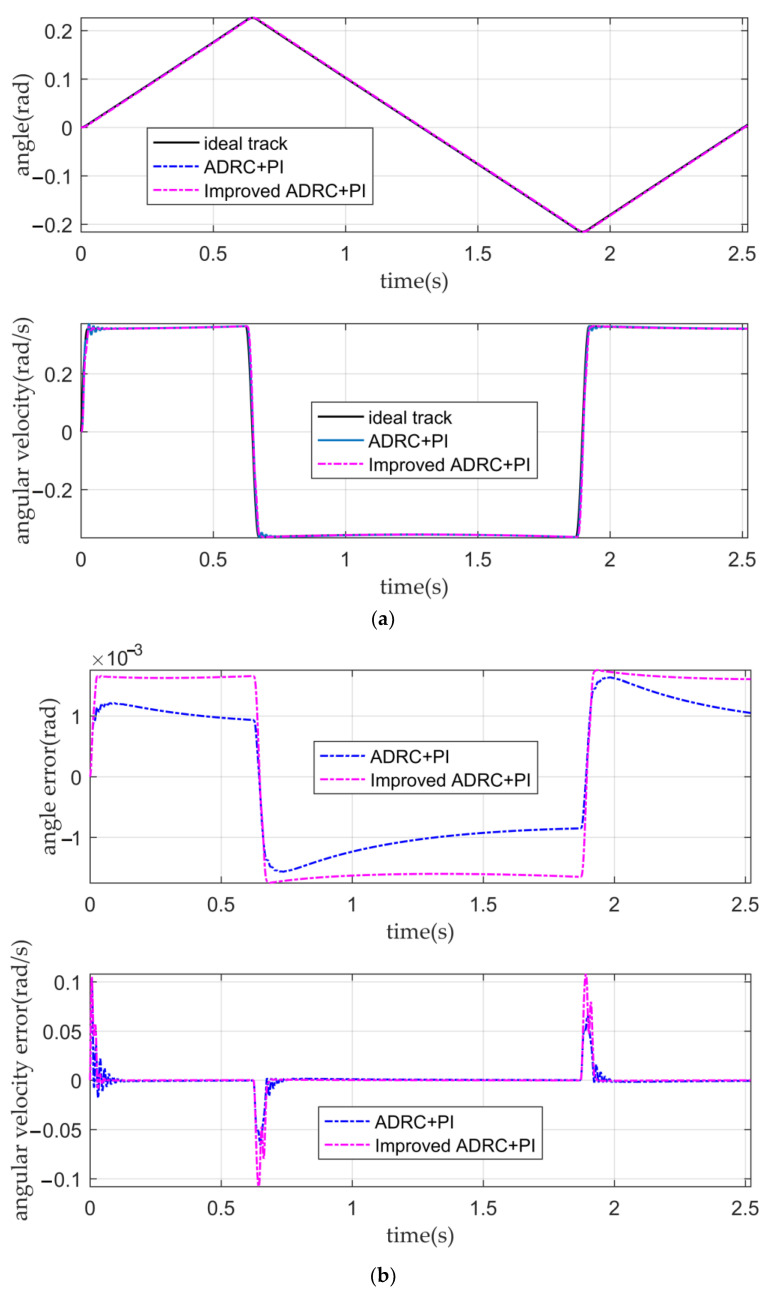
The tracking curves and error curves. (**a**) The angular displacement tracking curves and the angular velocity tracking curves of the conventional active disturbance rejection double closed-loop controller and the IADR-DCLC; (**b**) the angular displacement error curves and angular velocity error curves of the conventional active disturbance rejection double closed-loop controller and the IADR-DCLC.

**Figure 12:**
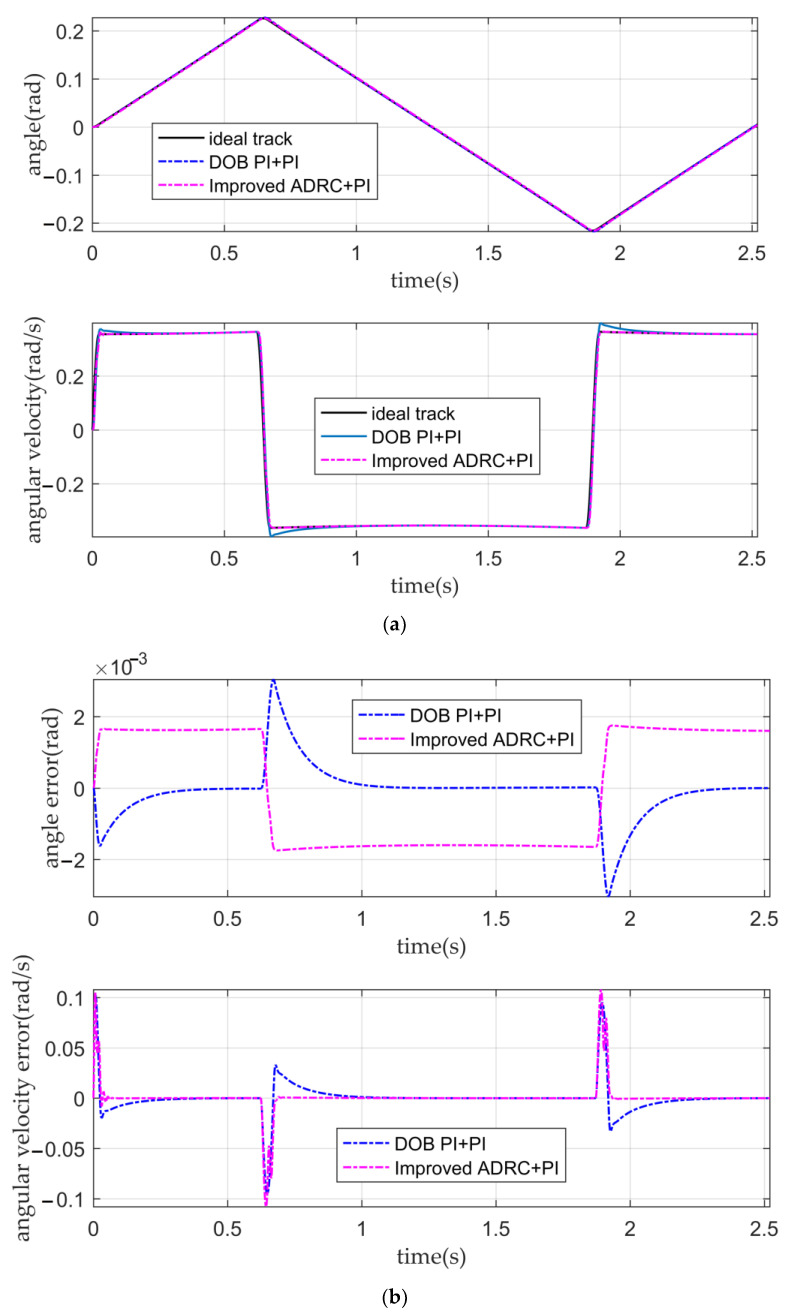
The tracking curves and the error curves. (**a**) The angular displacement tracking curves and the angular velocity tracking curves of the PI double closed-loop controller based on DOB and the IADR-DCLC; (**b**) the angular displacement error curves and the angular velocity error curves of the PI double closed-loop controller based on DOB and the IADR-DCLC.

When the input is the desired signal, the tracking curves and the error curves of the angular displacement and angular velocity of the five controllers are shown in Figure 10, Figure 11 and Figure 12, respectively.

We intercepted the data of the angular velocity and the angular displacement in the stable motion stage and calculated the optical-path scanning speed according to Equation (9). We calculated the ratio of the standard deviation of the optical-path scanning speed to the set optical-path scanning speed to measure the stability of the optical-path scanning speed.

It can be seen from Figure 10a,b and Table 4 that the classical PI double closed-loop controller, the fuzzy PI double closed-loop controller, and the IADR-DCLC have a good tracking ability for angular displacement, and the mean square error (MSE) of the angular displacement is to the order of 10^−3^. The tracking ability of the angular displacement of the fuzzy PI double closed-loop controller is slightly better than that of the IADR-DCLC. However, for the angular velocity, the tracking ability of the angular velocity of the classical PI double closed-loop controller and the fuzzy PI double closed-loop controller is poor. There is a large fluctuation of the angular velocity in the stable movement stage, resulting in the instability of the optical-path scanning speed, which cannot meet the requirement if the fluctuation of the optical-path scanning speed is less than 2% [28]. The stability of the optical-path scanning speed of the IADR-DCLC reaches 99.47%; that is, the fluctuation of the optical-path scanning speed is 0.53%, which meets the requirement that the fluctuation of the optical-path scanning speed of the moving mirror control system is less than 2%.

It can be seen from Figure 11a,b and Table 4 that when there is no disturbance in the system or the tracking ability of the conventional active disturbance rejection double closed-loop controller, and the IADR-DCLC for angular displacement and angular velocity is similar. Their angular displacement error is small, and their tracking ability of the angular velocity is good. Both controllers can ensure that the fluctuation of the optical-path scanning speed of the moving mirror control system is less than 2%. According to Figure 11b, in the initial stage of the simulation, the tracking error of the angular velocity of the IADR-DCLC is greater than that of the ADRC. The reason is that the actual angular velocity is directly used in the improved ADRC, while the actual angular displacement is used in the conventional ADRC [22]. The expected curve of the angular velocity is similar to the step signal in the initial stage, and there is a large error between the actual angular velocity used in the initial stage and the expected angular velocity. However, the initial error is negligible. When the system enters the stable motion stage, the error between the actual angular velocity used and the expected angular velocity is reduced so that the tracking error of the angular velocity of the IADR-DCLC in the stable motion stage is less than that of the conventional active disturbance rejection double closed-loop controller.

It can be seen from Figure 12a,b and Table 4 that the PI double closed-loop controller based on DOB has a good angular displacement tracking ability. Although the tracking ability of the angular velocity is weaker than that of the IADR-DCLC, it is also better than that of the classical PI double closed-loop controller and fuzzy PI double closed-loop controller, which meets the requirement that the fluctuation of the optical-path scanning speed of the moving mirror control system is less than 2%.

#### 3.2.3. Simulation of Controllers with Disturbance

The total disturbance observed by the ADRC is the superposition of the external disturbance and internal disturbance. The RT VCM will be affected by the uncertainty of the dynamic parameters and the external nonlinear disturbance in the movement process so that the total disturbance changes with the continuous movement of the RT VCM and the total disturbance can be regarded as a time-varying function [34]. In this study, we assumed that the time-varying function $f_{dist}(t)$ corresponding to the total disturbance is as follows:

(18) $$f_{dist}(t)=0.3\sin(2\pi t)+0.6\sin(\pi t)+0.2$$

**Figure 13:**
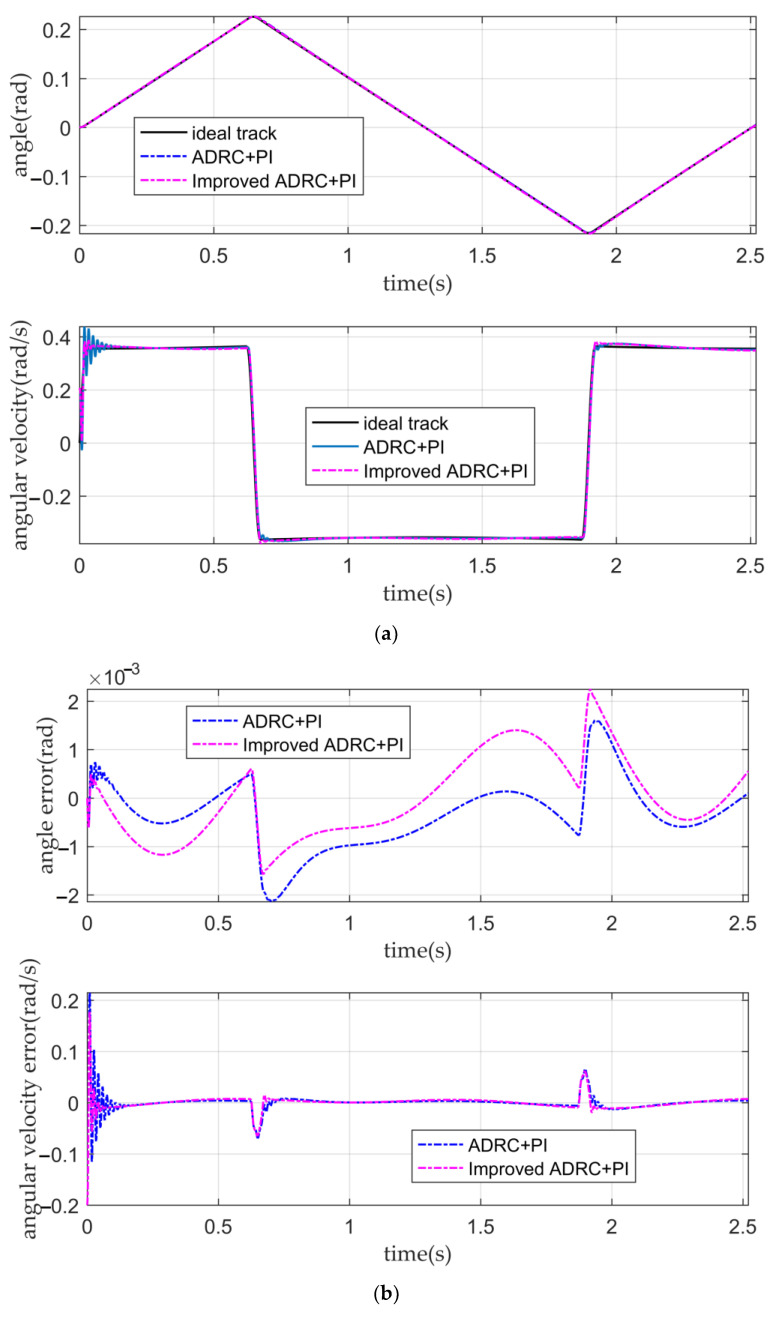
The tracking curves and error curves. (**a**) The angular displacement tracking curves and the angular velocity tracking curves of the conventional active disturbance rejection double closed-loop controller and the IADR-DCLC; (**b**) the angular displacement error curves and the angular velocity error curves of the conventional active disturbance rejection double closed-loop controller and the IADR-DCLC.

**Figure 14:**
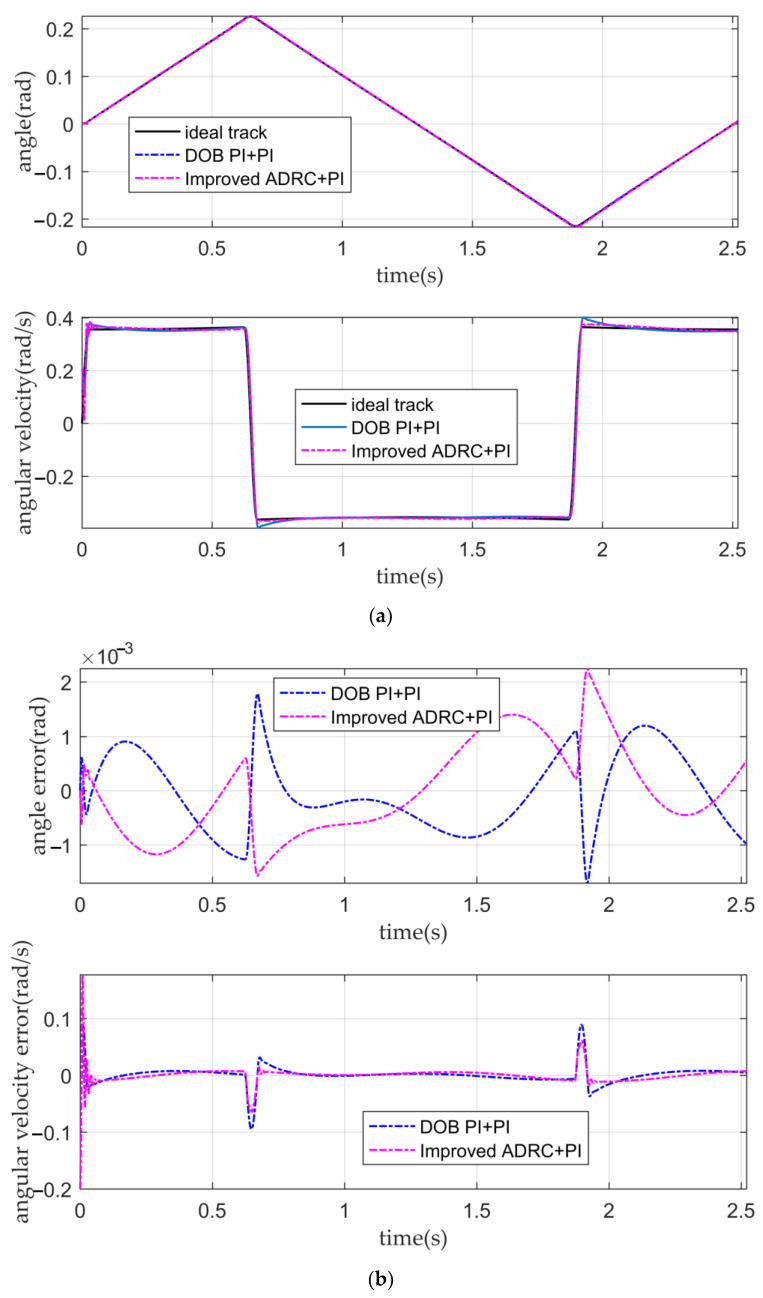
The tracking curves and the error curves. (**a**) The angular displacement tracking curves and the angular velocity tracking curves of the PI double closed-loop controller based on DOB and the IADR-DCLC; (**b**) the angular displacement error curves and the angular velocity error curves of the PI double closed-loop controller based on DOB and the IADR-DCLC.

Three controllers that can meet the requirements of the moving mirror control system without disturbance are tested. Keeping the parameters of the three controllers unchanged, the tracking curves and the error curves of the angular displacement and the angular velocity of the three controllers after the total disturbance are added to the system, as shown in Figure 13 and Figure 14, respectively.

Table 5 shows that the tracking ability of the angular displacement of the conventional active disturbance rejection double closed-loop controller and the IADR-DCLC does not decrease after adding nonlinear disturbance to the system. However, it can be seen from Figure 13b that the tracking ability of the angular displacement is still affected by the disturbance and shows a certain fluctuation. It can also be seen that the tracking ability of the angular velocity of the conventional active disturbance rejection double closed-loop controller is lower than that of the IADR-DCLC, which cannot meet the requirements of the moving mirror control system. The IADR-DCLC shows a good ability to suppress disturbance, and the stability of the optical-path scanning speed is still more than 98%.

It can be seen from Figure 14 and Table 5 that after adding disturbance, the PI double closed-loop controller based on DOB has a better angular displacement tracking ability than the IADR-DCLC, and the PI double closed-loop controller based on DOB also has a good anti-disturbance ability. However, the tracking ability of the angular velocity of the PI double closed-loop controller based on DOB is worse than that of the IADR-DCLC, which cannot meet the requirements of the system.

**Figure 15:**
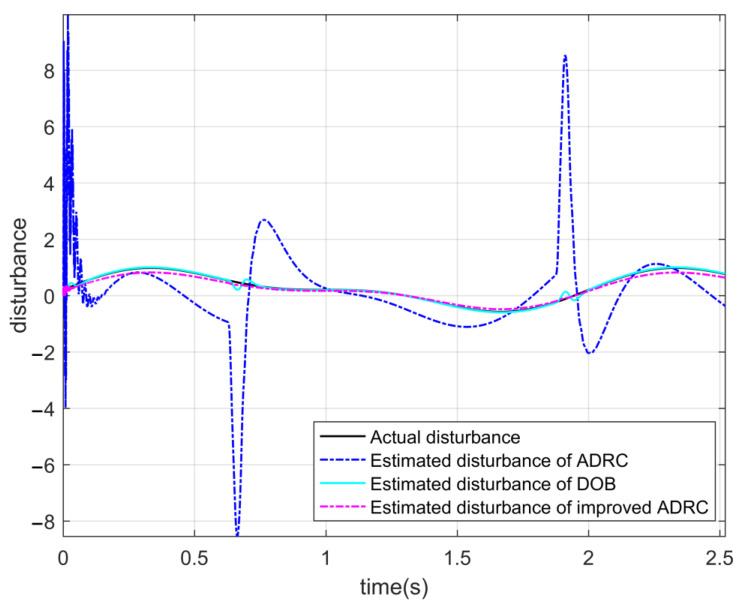
The observed disturbance curves of the three controllers.

The curves in Figure 15 are the disturbance curves observed by the three controllers. It can be seen that among the three controllers that the disturbance observed by the conventional active disturbance rejection double closed-loop controller is the worst; especially in the initial and commutation stages of the RT VCM, the error of disturbance is large, which seriously affects the performance of the controller. The disturbance observed by the PI double closed-loop controller based on DOB is also affected by the commutation of the RT VCM, and there is a certain error that affects the smooth operation of the RT VCM. The disturbance observed by the IADR-DCLC is very close to the disturbance added to the system and is not affected by the commutation of the RT VCM.

### 3.3. Analysis of Simulation Results

The simulation results show that when the disturbance is not considered, the five controllers can achieve good tracking performance for angular displacement. However, the tracking performance of the angular velocity of the five controllers is different; the tracking performance of the angular velocity of the classical PI double closed-loop controller and the fuzzy PI double closed-loop controller is poor, and the tracking performance of the angular velocity of the conventional active disturbance rejection double closed-loop controller, the PI double closed-loop controller based on DOB, and the IADR-DCLC is better than that of the classical PI double closed-loop controller and the fuzzy PI double closed-loop controller. Among the five controllers, the IADR-DCLC has the best tracking performance for angular velocity. Compared with the conventional active disturbance rejection double closed-loop controller, the IADR-DCLC introduces the actual angular velocity into the extended state observer, resulting in a large initial error of angular velocity in the initial stage of the RT VCM. However, the IADR-DCLC can quickly enter the stable operation state, and the impact of the initial error on the system is minimal.

When the time-varying disturbance is introduced into the system, the conventional active disturbance rejection double closed-loop controller, the PI double closed-loop controller based on DOB, and the IADR-DCLC show a certain anti-disturbance ability, of which the IADR-DCLC has the best anti-disturbance ability. The acceleration of the RT VCM in initial and commutation stages is relatively large, which will affect the tracking performance of the controllers and the disturbance observed by the controllers.

The conventional active disturbance rejection double closed-loop controller can observe the time-varying disturbance of the input, but there is a certain error between the observed disturbance and the actual disturbance, especially in the initial and commutation stages of the RT VCM, resulting in the poor tracking performance of the angular velocity of the controller. The disturbance error observed by the PI double closed-loop controller based on DOB is less than that of the conventional active disturbance rejection double closed-loop controller, but the observed disturbance is also not very ideal due to the commutation of the RT VCM. The IADR-DCLC proposed in this paper introduces the actual angular velocity into the extended state observer, which ensures the observed disturbance is fairly representative of the actual disturbance. Compared to the other controllers in this paper, it provides better compensation for the system and improves the control performance of the system. Under the simulation conditions in this paper, among the three controllers, only IADR-DCLC proposed in this paper meets the requirements of the moving mirror control system under the condition of adding disturbance to the system.

Therefore, it can be seen from the simulation results that the IADR-DCLC proposed in this paper has not only good tracking performance but also good anti-disturbance ability.

## 4. Conclusions

The RT VCM in the moving mirror control system is easily affected by its mechanical characteristics and the external environment, showing a certain nonlinearity. Aiming to address this problem, an IADR-DCLC is proposed in this paper. First, the mathematical model of the RT VCM was analyzed and established. Second, based on the requirements of the moving mirror control system for the stability of the optical-path scanning speed, the relationship between the optical-path scanning speed and the angular speed of the RT VCM was deduced. According to this relationship, the desired angular speed curve and the desired angular displacement curve of the RT VCM were planned. Finally, an improved ADRC was designed. The desired signal optimization was used to replace the tracking differentiator of the ADRC, which shortened the response time of the ADRC. The extended state observer of ADRC was improved, and the actual speed was introduced to ensure the disturbance observed by the observer is fairly representative of the actual disturbance. The simulation results show that the IADR-DCLC has a good tracking effect on the desired signal, can better observe and compensate for the disturbance, and has a good suppression effect on the internal and external disturbances of the system. Therefore, in the moving mirror control system, the IADR-DCLC can have a good control effect on the angular velocity and the angular displacement of the RT VCM.

## Figures and Tables

**Table 1:** The performance index of the moving mirror control system.

Parameter	Value
OPD velocity	0.064 m/s
Length of swing arm	45 mm
Scan cycle	2.52 s
Time of reversal	45 ms

**Table 2:** The parameters of the moving mirror control system.

Parameter	Value
Motor resistance	8.8 Ω
Motor inductance	4.2 mH
Sum of moments of inertia	2.4 × 10^−2^ kg × m^2^
Friction damping coefficient	0.68 N⋅s⋅m^−1^
Torque constant	0.35 Nm/A
Elastic damping coefficient	0.008 N⋅m^−1^

**Table 3:** The step response index.

Controller	Rise Time (s)	Overshoot	Steady-State Value
PI + PI	0.018	8.9%	1
Fuzzy PI + PI	0.016	8.0%	1
ADRC + PI	0.070	0.5%	1
DOB PI + PI	0.009	12.2%	1
The improved ADRC + PI	0.049	0.5%	1

**Table 4:** The tracking performance of five controllers.

Controller	MSE of Angular Displacement (10^−3^)	MSE of Angular Velocity (10^−2^)	MSE of Optical Path Scanning Speed (10^−3^)	Speed Stability
PI + PI	1.60	3.34	2.8	95.7%
Fuzzy PI + PI	1.40	3.04	2.4	96.2%
ADRC + PI	1.20	1.05	0.5	99.3%
DOB PI + PI	0.70	1.58	1.2	98.3%
The improved ADRC + PI	1.60	1.51	0.3	99.4%

**Table 5:** The tracking performance of the three controllers after adding disturbance.

Controller	MSE of Angular Displacement (10^−3^)	MSE of Angular Velocity (10^−2^)	MSE of Optical Path Scanning Speed (10^−3^)	Speed Stability
ADRC + PI	0.80	1.74	1.5	97.7%
DOB PI + PI	0.70	1.69	1.4	97.6%
The improved ADRC + PI	0.80	1.49	1.0	98.5%

## Data Availability

Not applicable.
